# 4-(5-Oxo-5*H*-1,2,4-dithia­zol-3-yl)phenyl 4-methyl­benzene­sulfonate

**DOI:** 10.1107/S1600536811055607

**Published:** 2012-01-18

**Authors:** Jian-Zhong Yang, Wei-Yi Pi, You-Fu Luo

**Affiliations:** a State Key Laboratory of Biotherapy, West China Hospital, Sichuan University, Chengdu 610041, People’s Republic of China

## Abstract

In the mol­ecular structure of the title compound, C_15_H_11_NO_4_S_3_, the 1,2,4-dithia­zolone and central benzene rings are approximately coplanar, making a dihedral angle of 3.08 (7)°. The central benzene ring and the 4-methyl­benzene ring subtend a dihedral angle of 57.47 (8)°. In the crystal, π–π stacking occurs between the central benzene ring and the 1,2,4-dithia­zolone ring of adjacent mol­ecules, which are aligned almost parallel, the centroid–centroid distance being 3.555 (7) Å.

## Related literature

For the synthesis of related compounds, see: Cho *et al.* (2003 ▶); Chen *et al.* (1996 ▶). For their biological activity, see: Iwakawa *et al.* (1994 ▶).
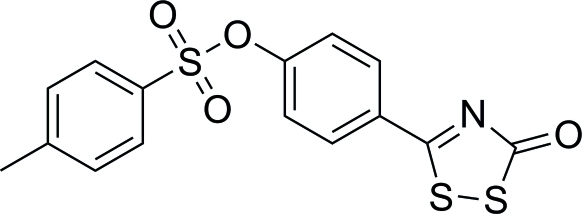


## Experimental

### 

#### Crystal data

C_15_H_11_NO_4_S_3_
*M* *_r_* = 365.43
Orthorhombic, 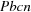

*a* = 30.2449 (9) Å
*b* = 7.0841 (3) Å
*c* = 14.6755 (5) Å
*V* = 3144.36 (18) Å^3^
*Z* = 8
Mo *K*α radiation
μ = 0.49 mm^−1^
*T* = 145 K
0.25 × 0.20 × 0.20 mm

#### Data collection

Agilent Xcalibur Eos diffractometer
Absorption correction: multi-scan (*CrysAlis PRO*; Agilent, 2010 ▶) *T*_min_ = 0.952, *T*_max_ = 1.000
6725 measured reflections
2775 independent reflections
2355 reflections with *I* > 2σ(*I*)
*R*_int_ = 0.022

#### Refinement

*R*[*F* ^2^ > 2σ(*F* ^2^)] = 0.032
*wR*(*F* ^2^) = 0.078
*S* = 1.04
2775 reflections
209 parameters
H-atom parameters constrained
Δρ_max_ = 0.41 e Å^−3^
Δρ_min_ = −0.29 e Å^−3^

### 

Data collection: *CrysAlis PRO* (Agilent, 2010 ▶); cell refinement: *CrysAlis PRO*; data reduction: *CrysAlis PRO*; program(s) used to solve structure: *SHELXS97* (Sheldrick, 2008 ▶); program(s) used to refine structure: *SHELXL97* (Sheldrick, 2008 ▶); molecular graphics: *OLEX2* (Dolomanov *et al.*, 2009 ▶); software used to prepare material for publication: *OLEX2*.

## Supplementary Material

Crystal structure: contains datablock(s) I, global. DOI: 10.1107/S1600536811055607/im2348sup1.cif

Structure factors: contains datablock(s) I. DOI: 10.1107/S1600536811055607/im2348Isup2.hkl

Supplementary material file. DOI: 10.1107/S1600536811055607/im2348Isup3.cml

Additional supplementary materials:  crystallographic information; 3D view; checkCIF report

## Figures and Tables

**Fig. 1.**
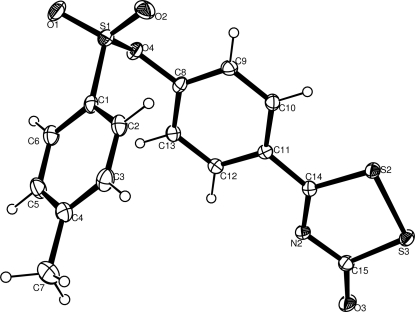
Molecular structure of the title compound with displacement ellipsoids drawn at the 30% probability level.

**Fig. 2.**
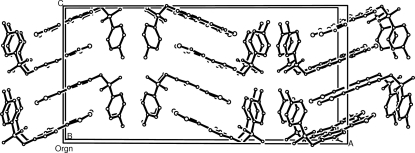
Crystal structure of the title compound.

## supplementary crystallographic information

### Comment

3*H*-1,2,4-dithiazol-3-one derivatives have been reported to posses antibacterial activity against a variety of bacteria and fungus strains including *Staphylococcus aureus*, *Pasteurella piscicida* and *Botlytis cinerea* (Iwakawa *et al.*, 1994).

The molecular structure of the title compound is shown in Fig. 1. The central benzene ring is twisted away from the planes of the 3*H*-1,2,4-dithiazol-3-one and 4-methylbenzene rings by 3.08 (7) and 57.47 (8)°, respectively. In the crystal structure, intermolecular π-π stacking between central benzene rings and 3*H*-1,2,4-dithiazol-3-one rings of adjacent molecules which are aligned almost parallel {centroid–centroid distance = 3.555 (7) Å} are observed (Fig. 2).

### Experimental

To a solution of 4-hydroxybenzothioamide (19.10 mmol) in chloroform (20 ml) at 273 K was added pyridine (3.70 ml, 45.84 mmol) dropwise over a period of 20 min and then *p*-toluenesulfonyl chloride (22.92 mmol) in small portions. This reaction mixture was stirred at room temperature for 12 h and diluted with dichloromethane and then 10% aqueous HCl. The separated organic layer was washed with 10% aqueous HCl, water and saturated aqueous NaCl. The organic phase was then dried over Na_2_SO_4_ and concentrated *in vacuo*. Recrystallization from EtOAc afforded 4-carbamothioylphenyl 4-methylbenzenesulfonate. To the solution of 4-carbamothioylphenyl 4-methylbenzenesulfonate (2 mmol) in THF (10 ml) was added chlorocarbonyl sulfenyl chloride (350 µ*L*, 4 mmol). The mixture was stirred at ambient temperature for 16 h. The solvent was removed *in vacuo* and the residue was purified by silica-gel column chromatography using EtOAc/hexane as eluent solvent system to get the title compound. Crystals suitable for X-ray analysis were obtained by slow evaporation from a solution in EtOAc at room temperature.

### Refinement

H atoms were positioned geometrically and refined using a riding model with C—H = 0.95 Å and *U*_iso_(H) = 1.2*U*_eq_(C) for aromatic H atoms and C—H = 0.98 Å and *U*_iso_(H) = 1.5*U*_eq_(C) for methyl H atoms.

### Crystal data

**Table:**

C_15_H_11_NO_4_S_3_	*D*_x_ = 1.544 Mg m^−^^3^
*M**_r_* = 365.43	Mo *K*α radiation, λ = 0.7107 Å
Orthorhombic, *P**b**c**n*	Cell parameters from 3356 reflections
*a* = 30.2449 (9) Å	θ = 2.9–28.8°
*b* = 7.0841 (3) Å	µ = 0.49 mm^−^^1^
*c* = 14.6755 (5) Å	*T* = 145 K
*V* = 3144.36 (18) Å^3^	Block, colorless
*Z* = 8	0.25 × 0.20 × 0.20 mm
*F*(000) = 1504	

### Data collection

**Table:**

Agilent Xcalibur Eos diffractometer	2775 independent reflections
Radiation source: Enhance (Mo) X-ray Source	2355 reflections with *I* > 2σ(*I*)
graphite	*R*_int_ = 0.022
Detector resolution: 16.0874 pixels mm^-1^	θ_max_ = 25.0°, θ_min_ = 3.0°
ω scans	*h* = −33→35
Absorption correction: multi-scan (CrysAlis PRO; Agilent, 2010)	*k* = −6→8
*T*_min_ = 0.952, *T*_max_ = 1.000	*l* = −10→17
6725 measured reflections	

### Refinement

**Table:**

Refinement on *F*^2^	Primary atom site location: structure-invariant direct methods
Least-squares matrix: full	Secondary atom site location: difference Fourier map
*R*[*F*^2^ > 2σ(*F*^2^)] = 0.032	Hydrogen site location: inferred from neighbouring sites
*wR*(*F*^2^) = 0.078	H-atom parameters constrained
*S* = 1.04	*w* = 1/[σ^2^(*F*_o_^2^) + (0.0338*P*)^2^ + 1.4416*P*] where *P* = (*F*_o_^2^ + 2*F*_c_^2^)/3
2775 reflections	(Δ/σ)_max_ = 0.001
209 parameters	Δρ_max_ = 0.41 e Å^−^^3^
0 restraints	Δρ_min_ = −0.29 e Å^−^^3^

### Special details

**Table:**

Experimental. CrysAlisPro, Agilent Technologies, Version 1.171.35.11 (release 16-05-2011 CrysAlis171 .NET) (compiled May 16 2011,17:55:39) Empirical absorption correction using spherical harmonics, implemented in SCALE3 ABSPACK scaling algorithm.
Geometry. All e.s.d.'s (except the e.s.d. in the dihedral angle between two l.s. planes) are estimated using the full covariance matrix. The cell e.s.d.'s are taken into account individually in the estimation of e.s.d.'s in distances, angles and torsion angles; correlations between e.s.d.'s in cell parameters are only used when they are defined by crystal symmetry. An approximate (isotropic) treatment of cell e.s.d.'s is used for estimating e.s.d.'s involving l.s. planes.
Refinement. Refinement of *F*^2^ against ALL reflections. The weighted *R*-factor *wR* and goodness of fit *S* are based on *F*^2^, conventional *R*-factors *R* are based on *F*, with *F* set to zero for negative *F*^2^. The threshold expression of *F*^2^ > σ(*F*^2^) is used only for calculating *R*-factors(gt) *etc*. and is not relevant to the choice of reflections for refinement. *R*-factors based on *F*^2^ are statistically about twice as large as those based on *F*, and *R*- factors based on ALL data will be even larger.

### Fractional atomic coordinates and isotropic or equivalent isotropic displacement parameters (Å^2^)

**Table:**

	*x*	*y*	*z*	*U*_iso_*/*U*_eq_	
S1	−0.163728 (17)	0.52253 (8)	0.56985 (4)	0.03114 (16)	
S2	0.060198 (17)	0.13631 (7)	0.67063 (4)	0.02683 (15)	
S3	0.094456 (17)	−0.09859 (7)	0.70721 (4)	0.02913 (15)	
O1	−0.20211 (5)	0.5638 (2)	0.51776 (12)	0.0445 (4)	
O2	−0.13702 (5)	0.6722 (2)	0.60383 (12)	0.0418 (4)	
O3	0.05762 (5)	−0.4287 (2)	0.68332 (12)	0.0405 (4)	
O4	−0.13468 (5)	0.3962 (2)	0.50138 (10)	0.0308 (4)	
N2	0.01393 (5)	−0.1767 (2)	0.63961 (12)	0.0252 (4)	
C1	−0.17634 (6)	0.3654 (3)	0.65777 (15)	0.0255 (5)	
C2	−0.15800 (7)	0.3921 (3)	0.74340 (16)	0.0312 (5)	
H2	−0.1378	0.4924	0.7540	0.037*	
C3	−0.16952 (7)	0.2713 (4)	0.81257 (16)	0.0379 (6)	
H3	−0.1574	0.2903	0.8716	0.045*	
C4	−0.19842 (7)	0.1220 (4)	0.79851 (17)	0.0388 (6)	
C5	−0.21578 (7)	0.0968 (3)	0.71170 (18)	0.0402 (6)	
H5	−0.2353	−0.0057	0.7007	0.048*	
C6	−0.20530 (7)	0.2173 (3)	0.64109 (16)	0.0324 (5)	
H6	−0.2177	0.1993	0.5822	0.039*	
C7	−0.21083 (9)	−0.0087 (5)	0.8753 (2)	0.0632 (9)	
H7A	−0.2430	−0.0261	0.8761	0.095*	
H7B	−0.2012	0.0460	0.9333	0.095*	
H7C	−0.1964	−0.1311	0.8664	0.095*	
C8	−0.09673 (6)	0.3042 (3)	0.53602 (14)	0.0249 (5)	
C9	−0.05942 (6)	0.4055 (3)	0.55942 (15)	0.0287 (5)	
H9	−0.0588	0.5390	0.5535	0.034*	
C10	−0.02302 (7)	0.3088 (3)	0.59164 (15)	0.0275 (5)	
H10	0.0028	0.3762	0.6091	0.033*	
C11	−0.02389 (6)	0.1121 (3)	0.59875 (13)	0.0218 (4)	
C12	−0.06182 (6)	0.0141 (3)	0.57370 (14)	0.0245 (4)	
H12	−0.0625	−0.1197	0.5780	0.029*	
C13	−0.09845 (7)	0.1102 (3)	0.54266 (14)	0.0264 (5)	
H13	−0.1245	0.0437	0.5261	0.032*	
C14	0.01431 (6)	0.0060 (3)	0.63420 (13)	0.0224 (4)	
C15	0.05148 (7)	−0.2615 (3)	0.67444 (15)	0.0282 (5)	

### Atomic displacement parameters (Å^2^)

**Table:**

	*U*^11^	*U*^22^	*U*^33^	*U*^12^	*U*^13^	*U*^23^
S1	0.0248 (3)	0.0286 (3)	0.0401 (3)	0.0061 (2)	0.0028 (2)	0.0039 (3)
S2	0.0256 (3)	0.0214 (3)	0.0335 (3)	0.0013 (2)	−0.0051 (2)	−0.0017 (2)
S3	0.0264 (3)	0.0249 (3)	0.0361 (3)	0.0036 (2)	−0.0070 (2)	−0.0011 (2)
O1	0.0302 (8)	0.0534 (11)	0.0498 (11)	0.0164 (8)	−0.0020 (8)	0.0137 (9)
O2	0.0383 (9)	0.0240 (8)	0.0631 (11)	−0.0013 (7)	0.0100 (9)	−0.0006 (8)
O3	0.0388 (9)	0.0220 (8)	0.0607 (11)	0.0036 (7)	−0.0145 (9)	0.0032 (8)
O4	0.0254 (7)	0.0375 (9)	0.0297 (8)	0.0089 (6)	−0.0002 (7)	0.0033 (7)
N2	0.0254 (9)	0.0224 (9)	0.0279 (10)	0.0009 (7)	−0.0009 (8)	0.0009 (8)
C1	0.0181 (10)	0.0261 (11)	0.0322 (11)	0.0028 (8)	0.0013 (9)	−0.0050 (9)
C2	0.0237 (11)	0.0336 (12)	0.0362 (13)	−0.0027 (9)	−0.0021 (10)	−0.0091 (10)
C3	0.0297 (12)	0.0543 (16)	0.0296 (12)	0.0033 (11)	−0.0023 (11)	−0.0029 (11)
C4	0.0259 (12)	0.0476 (15)	0.0428 (14)	0.0008 (10)	0.0059 (11)	0.0102 (12)
C5	0.0270 (12)	0.0368 (13)	0.0566 (16)	−0.0108 (10)	0.0000 (11)	0.0002 (12)
C6	0.0255 (11)	0.0347 (12)	0.0369 (13)	−0.0022 (10)	−0.0040 (10)	−0.0060 (11)
C7	0.0435 (15)	0.081 (2)	0.065 (2)	−0.0094 (15)	0.0105 (15)	0.0295 (17)
C8	0.0214 (10)	0.0319 (12)	0.0215 (10)	0.0066 (9)	0.0007 (9)	0.0013 (9)
C9	0.0280 (11)	0.0229 (11)	0.0350 (12)	0.0024 (9)	0.0036 (10)	0.0027 (10)
C10	0.0221 (10)	0.0275 (11)	0.0329 (12)	−0.0001 (9)	−0.0001 (9)	−0.0009 (10)
C11	0.0230 (10)	0.0237 (11)	0.0186 (10)	0.0030 (8)	0.0034 (8)	−0.0004 (8)
C12	0.0259 (10)	0.0230 (11)	0.0247 (10)	−0.0002 (8)	0.0027 (9)	−0.0016 (9)
C13	0.0238 (10)	0.0289 (12)	0.0266 (11)	−0.0004 (9)	0.0013 (9)	−0.0017 (9)
C14	0.0223 (10)	0.0263 (11)	0.0185 (10)	0.0002 (8)	0.0030 (8)	−0.0025 (9)
C15	0.0293 (11)	0.0252 (12)	0.0300 (11)	0.0001 (9)	−0.0028 (10)	−0.0007 (9)

### Geometric parameters (Å, °)

**Table:**

S1—O1	1.4204 (16)	C4—C7	1.506 (3)
S1—O2	1.4229 (16)	C5—H5	0.9500
S1—O4	1.6069 (15)	C5—C6	1.380 (3)
S1—C1	1.746 (2)	C6—H6	0.9500
S2—S3	2.0325 (7)	C7—H7A	0.9800
S2—C14	1.7506 (19)	C7—H7B	0.9800
S3—C15	1.803 (2)	C7—H7C	0.9800
O3—C15	1.206 (2)	C8—C9	1.381 (3)
O4—C8	1.414 (2)	C8—C13	1.379 (3)
N2—C14	1.297 (2)	C9—H9	0.9500
N2—C15	1.383 (3)	C9—C10	1.380 (3)
C1—C2	1.387 (3)	C10—H10	0.9500
C1—C6	1.388 (3)	C10—C11	1.398 (3)
C2—H2	0.9500	C11—C12	1.390 (3)
C2—C3	1.373 (3)	C11—C14	1.473 (3)
C3—H3	0.9500	C12—H12	0.9500
C3—C4	1.387 (3)	C12—C13	1.378 (3)
C4—C5	1.390 (3)	C13—H13	0.9500
			
O1—S1—O2	119.95 (10)	C4—C7—H7B	109.5
O1—S1—O4	103.00 (9)	C4—C7—H7C	109.5
O1—S1—C1	110.51 (10)	H7A—C7—H7B	109.5
O2—S1—O4	108.89 (9)	H7A—C7—H7C	109.5
O2—S1—C1	109.88 (10)	H7B—C7—H7C	109.5
O4—S1—C1	103.08 (9)	C9—C8—O4	120.89 (18)
C14—S2—S3	93.04 (7)	C13—C8—O4	116.98 (18)
C15—S3—S2	94.93 (7)	C13—C8—C9	122.11 (19)
C8—O4—S1	118.39 (13)	C8—C9—H9	120.7
C14—N2—C15	116.67 (17)	C10—C9—C8	118.61 (19)
C2—C1—S1	119.67 (16)	C10—C9—H9	120.7
C2—C1—C6	121.0 (2)	C9—C10—H10	119.8
C6—C1—S1	119.29 (17)	C9—C10—C11	120.38 (19)
C1—C2—H2	120.6	C11—C10—H10	119.8
C3—C2—C1	118.9 (2)	C10—C11—C14	121.35 (18)
C3—C2—H2	120.6	C12—C11—C10	119.56 (18)
C2—C3—H3	119.2	C12—C11—C14	119.07 (17)
C2—C3—C4	121.7 (2)	C11—C12—H12	119.9
C4—C3—H3	119.2	C13—C12—C11	120.30 (19)
C3—C4—C5	118.2 (2)	C13—C12—H12	119.9
C3—C4—C7	121.0 (2)	C8—C13—H13	120.5
C5—C4—C7	120.9 (2)	C12—C13—C8	119.03 (19)
C4—C5—H5	119.3	C12—C13—H13	120.5
C6—C5—C4	121.5 (2)	N2—C14—S2	120.98 (15)
C6—C5—H5	119.3	N2—C14—C11	121.59 (17)
C1—C6—H6	120.7	C11—C14—S2	117.42 (14)
C5—C6—C1	118.7 (2)	O3—C15—S3	119.25 (16)
C5—C6—H6	120.7	O3—C15—N2	126.36 (19)
C4—C7—H7A	109.5	N2—C15—S3	114.38 (15)
